# Nonlinear d^10^-ML_2_ Transition-Metal Complexes

**DOI:** 10.1002/open.201300009

**Published:** 2013-05-06

**Authors:** Lando P Wolters, F Matthias Bickelhaupt

**Affiliations:** [a] Department of Theoretical Chemistry and Amsterdam Center for Multiscale Modeling, VU UniversityDe Boelelaan 1083, 1081 HV Amsterdam (The Netherlands); [b] Institute for Molecules and Materials, Radboud University NijmegenHeyendaalseweg 135, 6525 AJ Nijmegen (The Netherlands)

**Keywords:** bond theory, density functional calculations, energy decomposition analysis, molecular geometry, transition-metal complexes, π backdonation

## Abstract

We have investigated the molecular geometries of a series of dicoordinated d^10^-transition-metal complexes ML_2_ (M=Co^−^, Rh^−^, Ir^−^, Ni, Pd, Pt, Cu^+^, Ag^+^, Au^+^; L=NH_3_, PH_3_, CO) using relativistic density functional theory (DFT) at ZORA-BLYP/TZ2P. Not all complexes have the expected linear ligand–metal–ligand (L–M–L) angle: this angle varies from 180° to 128.6° as a function of the metal as well as the ligands. Our main objective is to present a detailed explanation why ML_2_ complexes can become bent. To this end, we have analyzed the bonding mechanism in ML_2_ as a function of the L–M–L angle using quantitative Kohn–Sham molecular orbital (MO) theory in combination with an energy decomposition analysis (EDA) scheme. The origin of bent L–M–L structures is π backdonation. In situations of strong π backdonation, smaller angles increase the overlap of the ligand’s acceptor orbital with a higher-energy donor orbital on the metal-ligand fragment, and therefore favor π backdonation, resulting in additional stabilization. The angle of the complexes thus depends on the balance between this additional stabilization and increased steric repulsion that occurs as the complexes are bent.

## Introduction

**Figure 1:**
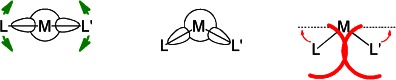
σ Donation has no preference (left, middle) whereas sterics favor linear L–M–L (right).

Dicoordinated d^10^-transition-metal complexes ML_2_ occur in numerous catalytic reaction mechanisms.^1^ These complexes, in general, have a linear geometry^2^–^5^ with a ligand–metal–ligand (L–M–L′) angle (or bite angle) of 180°, although exceptions^6^, ^7^ have been observed. This geometrical preference can be easily understood for a closed-shell d^10^ configuration. In most cases, the dominant bonding orbital interaction is σ donation from the ligand’s lone-pair orbitals into the empty metal (*n*+1)s atomic orbital (AO), which has a ligand–metal bond overlap that is independent of the L–M–L′ angle (see Figure 1).^8^ At the same time, the steric repulsion associated with a L⋅⋅⋅L′ overlap between the lone pairs (and other closed shells) of the two ligands yields a force that maximizes their mutual distance and thus yields the well-known linear L–M–L′ arrangement.

The same conclusion is obtained if one uses valence shell electron pair repulsion (VSEPR) theory adapted for treating transition-metal complexes,^9^, ^10^ or more sophisticated methods based on molecular orbital (MO) theory. Proceeding from the latter, one can deduce the preference for linear over bent ML_2_ complexes from the number of electrons in the valence orbitals and the dependence of the orbital energies on the geometrical parameter of interest (here, the L–M–L angle) in Walsh diagrams.^8^ These diagrams show again that dicoordinate d^10^-transition-metal complexes, for example, Ag(NH_3_)_2_^+^, adopt a linear geometry due to the significant destabilization of the metal d_xz_ AO by the ligand’s lone-pair orbitals in combination with steric repulsion between the latter upon bending (see below). Nearly all instances with substantial deviations of the L–M–L bite angle from linearity are complexes in which this distortion is imposed by the structural constraints in bidentate ligands in which a bridge or scaffold forces the two coordinating centers L towards each other.1b–d

In this work, we show that d^10^-ML_2_ complexes are not necessarily linear and may even have a pronounced intrinsic preference to adopt a nonlinear equilibrium geometry. To this end, we have investigated the molecular geometries and electronic structure of a series of d^10^-ML_2_ complexes (M=Co^−^, Rh^−^, Ir^−^, Ni, Pd, Pt, Cu^+^, Ag^+^, Au^+^; L=NH_3_, PH_3_, CO) using relativistic density functional theory (DFT). Simple d^10^-ML_2_ complexes are found with substantial deviations from linearity, featuring bite angles as small as 131° or even less. All that is necessary for bent d^10^-ML_2_ complexes to occur is sufficiently strong π backdonation. This emerges from our detailed metal–ligand bonding analyses in the conceptual framework of quantitative MO theory contained in Kohn–Sham DFT. The analyses explain the phenomenon and provide a tool for rationally tuning the bite angle. Based on our analyses, we can augment the text-book Walsh diagram for bending ML_2_ complexes involving only σ donation with an extended Walsh diagram that also includes π backbonding.

## Theoretical Methods

**Computational details**: All calculations were carried out using the Amsterdam Density Functional (ADF) program developed by Baerends and co-workers^11^–^13^ The numerical integration was performed using the procedure developed by te Velde et al.^14^ The molecular orbitals (MOs) were expanded in a large uncontracted set of Slater-type orbitals (STOs): TZ2P (no Gaussian functions are involved). The TZ2P basis set^15^ is of triple-*ζ* quality for all atoms and has been augmented with two sets of polarization functions, that is, 2p and 3d on H, 3d and 4f on C, N, O and P, 4p and 4f on Co, Ni, Cu, 5p and 4f on Rh, Pd and Ag and 6p and 5f on Ir, Pt and Au. An auxiliary set of s, p, d, f and g STOs was used to fit the molecular density and to represent the Coulomb and exchange potentials accurately in each self-consistent field (SCF) cycle. All electrons are included in the variational treatment (no frozen-core approximation used).

Equilibrium structures were obtained by optimizations using analytical gradient techniques.^16^ Geometries and energies were calculated at the BLYP level of the generalized gradient approximation (GGA): exchange is described by Slater’s Xα potential,^17^ with nonlocal corrections due to Becke^18^ added self-consistently, and correlation is treated using the gradient-corrected functional of Lee, Yang and Parr.^19^ Scalar relativistic effects were accounted for using the zeroth-order regular approximation (ZORA).^20^ This approach has been extensively tested and was shown to agree well with high-level coupled-cluster reference data.^21^ Energy minima have been verified through vibrational analysis.^22^ All minima were found to have zero imaginary frequencies. The PyFrag program was used to facilitate the analyses of the bonding mechanism as a function of the L–M–L angle.^23^

**Bond energy analysis**: The bond energy Δ*E* is decomposed into the strain energy Δ*E*_strain_, that is associated with the geometrical deformation of the fragments as the bond formation takes place, plus the actual interaction energy Δ*E*_int_ between the deformed fragments [Equation (1)].


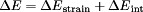
(1)

The interaction energy Δ*E*_int_(*ζ*) between two molecular fragments is analyzed as a function of the bite angle *ζ* in the conceptual framework provided by the Kohn–Sham MO method.^24^ To this end, it is decomposed in three physically meaningful terms [Eq. (2)] using a quantitative energy decomposition scheme developed by Ziegler and Rauk.^25^


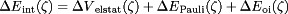
(2)

The term Δ*V*_elstat_ corresponds to the classical electrostatic interaction between the unperturbed charge distributions *ρ*_A_(*r*)+*ρ*_B_(*r*) of the prepared or deformed fragments A and B (see below for definition of the fragments) that adopt their positions in the overall molecule AB, and is usually attractive. The Pauli repulsion term Δ*E*_Pauli_ comprises the destabilizing interactions between occupied orbitals and is responsible for the steric repulsion. This repulsion is caused by the fact that two electrons with the same spin cannot occupy the same region in space. It arises as the energy change associated with the transition from the superposition of the unperturbed electron densities *ρ*_A_(*r*)+*ρ*_B_(*r*) of the geometrically deformed but isolated fragments A and B, to the wavefunction Ψ^0^=*N Â* [Ψ_A_ Ψ_B_], that properly obeys the Pauli principle through explicit antisymmetrization (*Â* operator) and renormalization (*N* constant) of the product of fragment wavefunctions (see Ref. 24 for an exhaustive discussion). The orbital interaction Δ*E*_oi_ accounts for charge transfer (interaction between occupied orbitals on one fragment with unoccupied orbitals on the other fragment, including the HOMO–LUMO interactions) and polarization (empty-occupied orbital mixing on one fragment due to the presence of another fragment). It can be further divided into contributions from each irreducible representation *Γ* of the interacting system [Eq. (3)].


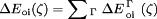
(3)

## Results and Discussion

### Structure and energetics

**Figure 2:**
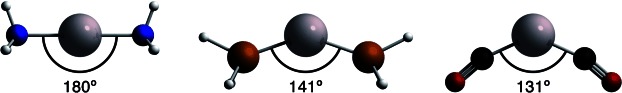
Equilibrium geometries computed at ZORA-BLYP/TZ2P. From left to right: Rh(NH_3_)_2_^−^, Rh(PH_3_)_2_^−^ and Rh(CO)_2_^−^.

Structural and energetic data emerging from our ZORA-BLYP/TZ2P computations are collected in Tables 1–4. Most ML_2_ complexes have a linear L–M–L angle, which leads to either *D*_3*h*_-symmetric complexes M(NH_3_)_2_ and M(PH_3_)_2_ or *D*_∞h_-symmetric complexes M(CO)_2_. However, numerous significantly smaller angles appear throughout Table 1 as well, where the symmetry of the complexes is lowered to *C*_2*v*_. For instance, the complexes become increasingly bent when the ligands are varied along NH_3_ (a strong σ donor), PH_3_ (a σ donor and π acceptor) and CO (a strong π acceptor). This is most clearly seen for the group 9 complexes, where, for example, the angle decreases along Rh(NH_3_)_2_^−^, Rh(PH_3_)_2_^−^ and Rh(CO)_2_^−^ from 180.0° to 141.2° and 130.8° (Figure 2). In a later section, we will show that the π-backbonding properties of the complexes constitute a prominent part of the explanation of why d^10^-ML_2_ complexes can adopt nonlinear geometries. The increasingly strong π backbonding along this series also results in stronger metal–ligand bonds (see Table 2 for bond dissociation energies (BDEs) and Table 3 for energy decomposition analyses (EDA) results for ML complexes).

**Table 1:** L–M–L angle [°] and linearization energy Δ*E*_lin_ [kcal mol^−1^] in dicoordinate d^10^-ML_2_ complexes^[a]^

Group 9			Group 10			Group 11		
	L–M–L	Δ*E*_lin_^[b]^		L–M–L	Δ*E*_lin_^[b]^		L–M–L	Δ*E*_lin_^[b]^
Co(NH_3_)_2_^−^	180.0	0	Ni(NH_3_)_2_	180.0	0	Cu(NH_3_)_2_^+^	180.0	0
Co(PH_3_)_2_^−^	131.8	6.4	Ni(PH_3_)_2_	180.0	0	Cu(PH_3_)_2_^+^	180.0	0
Co(CO)_2_^−^	128.6	19.9	Ni(CO)_2_	144.5	2.1	Cu(CO)_2_^+^	180.0	0
Rh(NH_3_)_2_^−^	180.0	0	Pd(NH_3_)_2_	180.0	0	Ag(NH_3_)_2_^+^	180.0	0
Rh(PH_3_)_2_^−^	141.2	2.0	Pd(PH_3_)_2_	180.0	0	Ag(PH_3_)_2_^+^	180.0	0
Rh(CO)_2_^−^	130.8	10.2	Pd(CO)_2_	155.6	0.5	Ag(CO)_2_^+^	180.0	0
Ir(NH_3_)_2_^−^	180.0	0	Pt(NH_3_)_2_	180.0	0	Au(NH_3_)_2_^+^	180.0	0
Ir(PH_3_)_2_^−^	144.1	2.4	Pt(PH_3_)_2_	180.0	0	Au(PH_3_)_2_^+^	180.0	0
Ir(CO)_2_^−^	134.2	13.4	Pt(CO)_2_	159.0	0.6	Au(CO)_2_^+^	180.0	0

[a] Computed at ZORA-BLYP/TZ2P. [b] Relative energy of the linear ML_2_ complex relative to its equilibrium geometry.

**Table 2:** M–L bond length [Å] and BDE [kcal mol^−1^] in monocoordinate d^10^-ML and dicoordinate d^10^-ML_2_ complexes^[a]^

	M–L	BDE		M–L	BDE		M–L	BDE
CoNH_3_^−[b,c]^	1.845	217.1	NiNH_3_^[c]^	1.827	77.0	CuNH_3_^+^	1.911	70.0
CoPH_3_^−[b,c]^	1.971	240.6	NiPH_3_^[c]^	1.979	88.0	CuPH_3_^+^	2.163	68.7
CoCO^−[b,c]^	1.630	280.6	NiCO^[c]^	1.663	109.3	CuCO^+^	1.833	50.2
Co(NH_3_)_2_^−[b,c]^	1.908	24.0	Ni(NH_3_)_2_^[c]^	1.888	36.2	Cu(NH_3_)_2_^+^	1.919	61.1
Co(PH_3_)_2_^−[c]^	2.051	48.2	Ni(PH_3_)_2_^[c]^	2.108	36.3	Cu(PH_3_)_2_^+^	2.232	48.0
Co(CO)_2_^−[c]^	1.715	76.3	Ni(CO)_2_^[c]^	1.765	48.6	Cu(CO)_2_^+^	1.882	45.0
RhNH_3_^−[c]^	2.001	55.5	PdNH_3_	2.115	21.6	AgNH_3_^+^	2.212	48.7
RhPH_3_^−[c]^	2.068	89.9	PdPH_3_	2.172	39.4	AgPH_3_^+^	2.415	47.9
RhCO^−[c]^	1.750	122.0	PdCO	1.861	47.4	AgCO^+^	2.137	28.4
Rh(NH_3_)_2_^−[c]^	2.089	22.6	Pd(NH_3_)_2_	2.106	28.6	Ag(NH_3_)_2_^+^	2.172	45.2
Rh(PH_3_)_2_^−[c]^	2.196	38.2	Pd(PH_3_)_2_	2.287	28.6	Ag(PH_3_)_2_^+^	2.444	38.1
Rh(CO)_2_^−[c]^	1.866	58.1	Pd(CO)_2_	1.949	34.7	Ag(CO)_2_^+^	2.113	30.7
IrNH_3_^−[c]^	1.967	85.0	PtNH_3_^[c]^	1.981	50.1	AuNH_3_^+^	2.085	71.4
IrPH_3_^−[c]^	2.056	126.5	PtPH_3_^[c]^	2.095	77.3	AuPH_3_^+^	2.240	84.2
IrCO^−[c]^	1.734	166.3	PtCO^[c]^	1.776	87.9	AuCO^+^	1.927	55.0
Ir(NH_3_)_2_^−[b,c]^	2.071	23.6	Pt(NH_3_)_2_^[c]^	2.061	41.6	Au(NH_3_)_2_^+^	2.088	64.6
Ir(PH_3_)_2_^−[c]^	2.190	44.1	Pt(PH_3_)_2_^[c]^	2.249	38.7	Au(PH_3_)_2_^+^	2.351	52.6
Ir(CO)_2_^−[c]^	1.854	66.3	Pt(CO)_2_^[c]^	1.911	47.1	Au(CO)_2_^+^	2.002	40.4

[a] Computed at ZORA-BLYP/TZ2P. Bond dissociation energies (BDEs) are given for the complexes in the electronic configuration corresponding to a d^10^s^0^ electron configuration and relative to closed-shell d^10^s^0^ metal atoms. [b] The d^10^s^0^-type configuration is an excited state of the complex. [c] The d^10^s^0^ configuration is an excited state of the atomic metal fragment.

**Table 3:** Energy decomposition analyses [kcal mol^−1^] and orbital energies *ε* [eV] for the metal–ligand bonds in monoligated transition-metal complexes M–L^[a]^

ML	Δ*E*	Δ*E*_int_	Δ*V*_elstat_	Δ*E*_Pauli_	Δ*E*_oi_	Δ 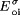	Δ 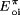 ^[b]^	*ε*[d_σ_]	*ε*[d_π_]	*ε*[d_δ_]
CoNH_3_^−^	−217.1	−218.4	−110.0	166.3	−274.7	−241.8	−32.9	+1.84	+2.91	+3.99
CoPH_3_^−^	−240.6	−241.7	-197.9	204.5	−248.2	−123.9	−124.4	+1.67	+1.81	+3.38
CoCO^−^	−280.6	−286.4	−233.4	274.5	−327.5	−141.7	−185.8	+1.34	+1.17	+3.20
RhNH_3_^−^	−55.5	−56.2	−143.2	202.1	−115.1	−110.8	−4.3	+1.72	+1.83	+2.53
RhPH_3_^−^	−89.9	−90.3	−269.7	311.7	−132.3	−61.7	−70.6	+1.49	+0.91	+2.20
RhCO^−^	−122.0	−126.0	−273.3	364.1	−216.8	−96.7	−120.1	+1.05	−0.09	+1.56
IrNH_3_^−^	−85.0	−85.8	−196.9	268.9	−157.8	−142.9	−14.9	+1.54	+2.16	+2.91
IrPH_3_^−^	−126.5	−127.2	−349.2	396.0	−174.1	−85.9	−88.2	+1.18	+0.73	+2.28
IrCO^−^	−166.3	−171.3	−353.5	461.5	−279.2	−129.6	−149.7	+0.63	−0.26	+1.68
NiNH_3_	−77.0	−77.3	−116.2	139.8	−100.8	−94.5	−6.3	−3.28	−2.99	−2.21
NiPH_3_	−88.0	−88.7	−161.3	173.3	−100.7	−50.8	−49.9	−3.79	−3.93	−2.90
NiCO	−109.3	−110.4	−171.6	210.3	−149.1	−60.4	−88.7	−4.89	−5.40	−4.14
PdNH_3_	−21.6	−21.7	−88.0	105.1	−38.8	−34.5	−4.4	−3.46	−3.81	−3.47
PdPH_3_	−39.4	−39.8	−166.2	190.3	−63.8	−35.3	−28.5	−4.49	−5.29	−4.56
PdCO	−47.4	−47.8	−161.4	213.3	−99.7	−48.0	−51.8	−5.28	−6.48	−5.53
PtNH_3_	−50.1	−50.4	−170.1	211.4	−91.7	−82.0	−9.7	−4.19	−4.46	−3.72
PtPH_3_	−77.3	−78.9	−273.9	310.3	−115.3	−70.5	−44.8	−4.92	−5.72	−4.53
PtCO	−87.9	−88.7	−271.6	356.9	−174.0	−91.6	−82.4	−5.97	−7.28	−5.77
CuNH_3_^+^	−70.0	−70.1	−104.5	86.0	−51.7	−41.9	−9.8	−11.80	−12.13	−12.02
CuPH_3_^+^	−68.7	−73.5	−101.7	94.0	−65.8	−51.8	−14.0	−11.99	−12.44	−12.15
CuCO^+^	−50.2	−50.3	−89.8	100.7	−61.2	−38.8	−22.4	−13.7	−14.28	−13.90
AgNH_3_^+^	−48.7	−48.7	−73.3	58.8	−34.2	−28.5	−5.8	−12.56	−13.60	−13.57
AgPH_3_^+^	−47.9	−51.8	−84.3	81.3	−48.8	−39.9	−8.9	−12.41	−13.67	−13.85
AgCO^+^	−28.4	−28.6	−59.1	67.2	−36.7	−26.2	−10.6	−14.08	−15.07	−14.86
AuNH_3_^+^	−71.4	−71.6	−124.8	123.2	−70.0	−60.3	−9.7	−12.49	−13.32	−12.92
AuPH_3_^+^	−84.2	−91.0	−177.9	187.2	−100.3	−80.9	−19.4	−12.52	−13.70	−13.06
AuCO^+^	−55.0	−55.1	−149.0	188.4	−94.5	−64.9	−29.7	−14.20	−15.53	−14.73

[a] Computed at ZORA-BLYP/TZ2P. See [Eqs. (1)–(3)]. [b] Also includes small contributions from δ orbital interactions, which can only be separated for *C*_∞v_-symmetric MCO complexes. There, the δ term amounts at most to 3.5 % of the π term.

**Table 4:** Ligand orbital energies *ε* [eV] and proton affinities [kcal mol^−1^]^[a]^

	*ε*(LP)	*ε*(π*)	PA
NH_3_	−6.05	+1.42	+201.4
PH_3_	−6.63	−0.24	+185.2
CO	−8.93	−1.92	+141.5

[a] Computed at ZORA-BLYP/TZ2P. LP: lone pair, π*: acceptor orbital. Proton affinities (PA) from enthalpies at 298.15 K and 1 atm.

The extent of bending systematically decreases when the π-backbonding capability of the metal center decreases from the group 9 anions, via neutral group 10 atoms, to the group 11 cations. This is clearly displayed by the series of isoelectronic complexes Rh(CO)_2_^−^, Pd(CO)_2_ and Ag(CO)_2_^+^ along which the L–M–L angle increases from 130.8° to 155.6° to 180° (Table 1). The data in Table 3 for the corresponding monocoordinate RhCO^−^, PdCO and AgCO^+^ nicely show how along this series the distortive π-orbital interactions Δ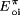 indeed become weaker, from −120 to −51 to −11 kcal mol^−1^, respectively. In the case of group 9 metals, both phosphine and carbonyl complexes are bent, whereas, for group 10 metals, only the carbonyl complexes deviate from linearity. Complexes with a metal center from group 11 all have a linear L–M–L configuration. The reduced π backbonding also leads to weaker metal–ligand bonds. For the cationic metal centers, for which π backdonation plays a much smaller role, the metal–ligand BDEs decrease in the order NH_3_>PH_3_>CO (see Table 2). This trend originates directly from the σ-donating capabilities of the ligands as reflected by the energy of the lone-pair orbital *ε*(LP), which decreases in this order (see Table 4). Note that, for the same reason, the basicity of the ligand as measured by the proton affinity (PA) decreases along NH_3_>PH_3_>CO.^26^ For the anionic group 9 metal centers, the opposite order is found, that is, metal–ligand BDEs decrease in the order CO>PH_3_>NH_3_, following the π-accepting capabilities of the ligands.

Linearity also increases if one descends in a group. For example, from Ni(CO)_2_ to Pd(CO)_2_ to Pt(CO)_2_, the L–M–L angle increases from 144.5° to 155.6° to 159.0°. Interestingly, this last trend is opposite to what one would expect proceeding from a steric model. If one goes from a larger to a smaller metal center, that is, going up in a group, the ligands are closer to each other and thus experience stronger mutual steric repulsion. But instead of becoming more linear to avoid such repulsion, the complexes bend even further in the case of the smaller metal. For example, when the palladium atom in Pd(CO)_2_ is replaced by a smaller nickel atom, the L–M–L angle decreases from 155.6° in Pd(CO)_2_ to 144.5° in Ni(CO)_2_. Later on, we show that this seemingly counterintuitive trend also originates from enhanced π backbonding which dominates the increased steric repulsion.

### General bonding mechanism

**Figure 3:**
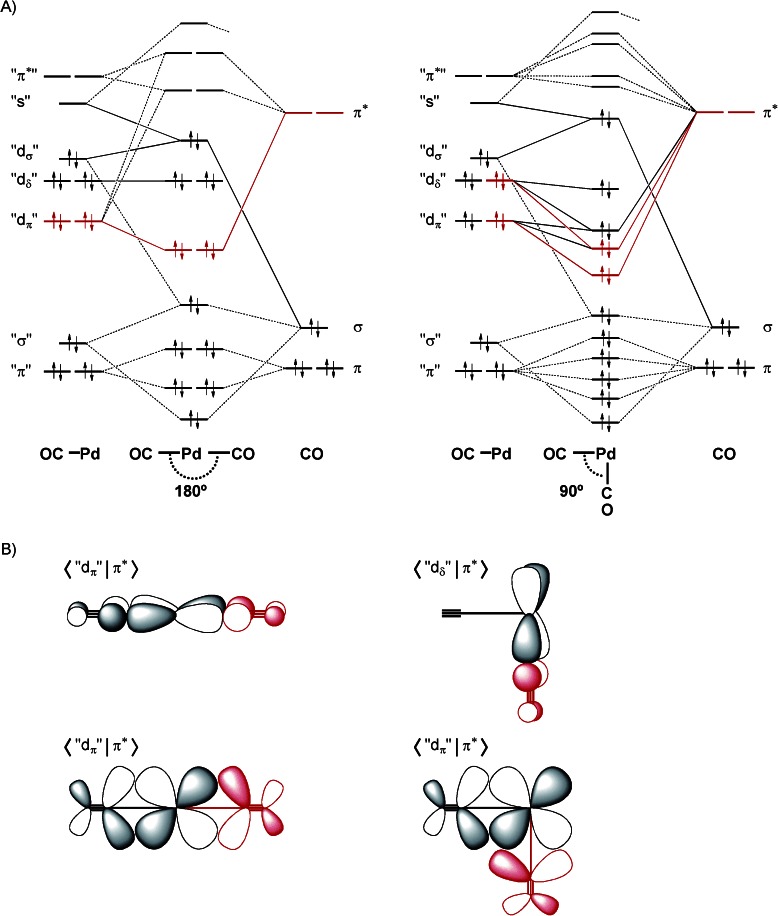
A) Schematic MO diagrams for the bonding mechanism between PdCO and CO in linear Pd(CO)_2_ (left) and at a L–M–L angle of 90° (right): dominant interactions (—), other interactions (- - - -), π backbonding (—). B) Schematic representation of the bonding overlaps of the donating orbital on PdCO (black) with the π-accepting orbital on the second CO ligand (red).

The bending of our model complexes can be understood in terms of a monocoordinate complex to which a second ligand is added either in a linear or a bent arrangement, ML+L→ML_2_ (see below). Using Pd(CO)_2_ as an example, we start from a PdCO fragment, and consider the addition of the second CO ligand both at a 180° angle and a 90° angle. Our Kohn–Sham MO analyses show that, in PdCO, the degeneracy of the five occupied d orbitals on palladium is lowered by interactions with the ligand (see Figure 3). Choosing the M–L bond along the *z* axis, the d_xz_ and d_yz_ orbitals act as donor orbitals for π backdonation into the two π*-acceptor orbitals on the CO ligand, resulting in two stabilized “d_π_” orbitals at −6.5 eV (value not shown in Figure 3). The d_xy_ and d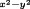 (or “d_δ_”) orbitals at −5.5 eV do not overlap and interact with the ligand. The d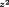 orbital is destabilized due to the antibonding overlap with the lone pair on the ligand, resulting in a “d_σ_” orbital that is relatively high in energy, at −5.3 eV.

When the second CO ligand coordinates opposite the first one (i.e., in a linear L–M–L arrangement), its π*-acceptor orbitals interact with the d_π_ orbitals on the PdCO fragment. The latter are already considerably stabilized by π backdonation to the first CO ligand (Figure 3 B, left). When, instead, the second ligand is added at an angle of 90°, its π* orbitals overlap with only one d_π_ orbital, and with one d_δ_ orbital (Figure 3 B, right). This d_δ_ orbital is essentially a pure metal d orbital that has *not* yet been stabilized by any coordination bond. Consequently, this orbital has a higher energy and is, therefore, a more capable donor orbital for π backdonation into the π* orbital of the second CO ligand. This results in a stronger, more stabilizing donor–acceptor interaction of this pair of orbitals in the 90° (Figure 3 A, right) than in the 180° ML_2_ geometry (Figure 3 A, left: cf. red-highlighted π interactions). σ-Donation interactions are affected less by bending. It is therefore π backdonation that favors bending. The more detailed energy decomposition analyses in the following sections consolidate this picture.

### Bonding mechanism: Variation of ligands

**Figure 4:**
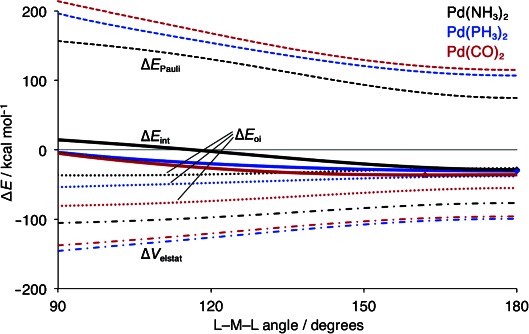
Energy decomposition analysis [Eq. (2)] of the interaction between PdL and L in dicoordinated palladium complexes PdL_2_ as a function of the L–M–L angle (L=NH_3_, PH_3_, CO).

**Figure 5:**
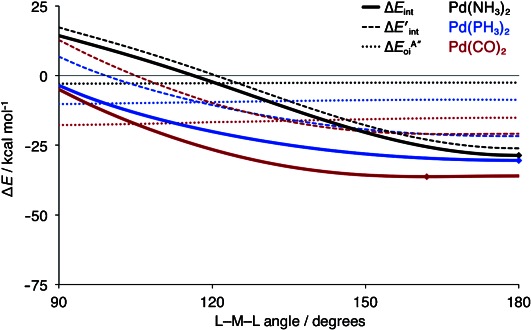
Energy decomposition analysis [Eq. (4)] of the interaction between PdL and L in dicoordinated palladium complexes PdL_2_ as a function of the L–M–L angle (L=NH_3_, PH_3_, CO).

**Figure 6:**
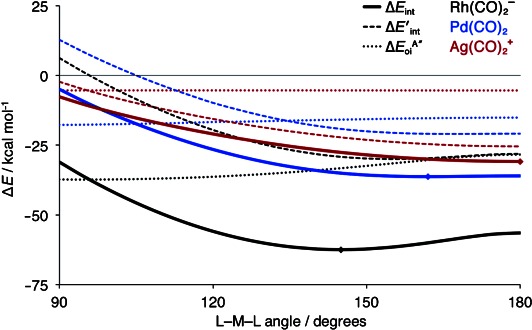
Energy decomposition analysis [Eq. (4)] of the interaction between MCO and CO in dicarbonyl-transition-metal complexes M(CO)_2_ as a function of the L–M–L angle (M=Rh^−^, Pd, Ag^+^).

**Figure 7:**
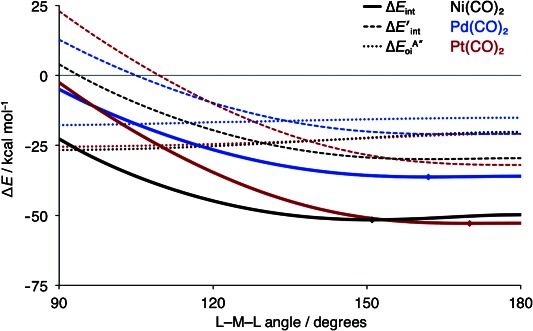
Energy decomposition analysis [Eq. (4)] of the interaction between MCO and CO in dicarbonyl-transition-metal complexes M(CO)_2_ as a function of the L–M–L angle (M=Ni, Pd, Pt).

To understand the trends in nonlinearity of our ML_2_ complexes (see above and Table 1), we have quantitatively analyzed the metal–ligand bonding between ML and the second ligand L as a function of the L–M–L angle. The results are collected in Table 2 and displayed in Figure 4–7. Most of our model complexes have a d^10^-type ground-state configuration but not all of them, as indicated in detail in Table 2. Yet, *all* model systems discussed here have been kept in d^10^-configuration, to achieve a consistent comparison and because, on the longer term, we are interested in understanding more realistic dicoordinated d^10^-transition-metal complexes that feature, for example, as catalytically active species in metal-mediated bond activation. We start in all cases from the optimal *linear* ML_2_ structure (i.e., the complex optimized in either *D*_∞h_ or *D*_3*h*_ symmetry) and then analyze the bonding between ML and L′ as a function of the L–M–L angle, from 180° to 90°, while keeping all other geometry parameters frozen. The analyses were done in *C*_s_ symmetry, bending the complexes in the mirror plane, with the out-of-plane hydrogen atoms of M(NH_3_)_2_ and M(PH_3_)_2_ towards each other. Thus, we are able to separate the orbital interactions symmetric to the mirror plane (A′ irrep) from the orbital interactions asymmetric to the mirror plane (A″ irrep): Δ*E*_oi_(*ζ*)= 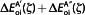 [Eq. (3)]. The use of frozen fragment geometries allows us to study purely how the interaction energy changes as the angle is varied, without any perturbation due to geometrical relaxation. Therefore, any change in Δ*E* stems exclusively from a change in Δ*E*_int_=Δ*V*_elstat_+Δ*E*_Pauli_+Δ
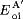
+Δ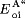. Note that rigid bending of the linearly optimized L–M–L complexes causes minima on the energy profiles to shift to larger angles than in fully optimized complexes, but this does not alter any relative structural or energy order.

In Figure 4, we show the energy decomposition analyses [Eq. (2)] and how they vary along the palladium complexes Pd(NH_3_)_2_, Pd(PH_3_)_2_ and Pd(CO)_2_. Upon bending the LM–L′ complex from 180° to 90°, the average distance between the electron density on LM and the nuclei of L′ decreases (the Pd–P distance however remains constant), which results in a more stabilizing electrostatic attraction Δ*V*_elstat_. Likewise, the Pauli repulsion Δ*E*_Pauli_ increases because of a larger overlap of the lone pair on L′ with the d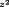-derived d_σ_ orbital on the ML fragment. The latter is the antibonding combination of the metal d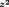 orbital and the ligand lone pair, with a fair amount of metal s character admixed in an L–M bonding fashion. The resulting hybrid orbital is essentially the d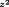 orbital with a relatively large torus. The increase in Pauli repulsion that occurs as the L–M–L′ angle decreases stems largely from the overlap of the lone pair on the second ligand L′ with this torus. For Pd(CO)_2_ for example, the overlap of the L′ lone pair with the d_σ_ hybrid orbital on ML increases from 0.05 to 0.28 upon bending from 180° to 90°. We note that this repulsion induces a secondary relaxation, showing up as a stabilizing Δ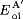, by which it is largely canceled again. The mechanism through which this relief of Pauli repulsion happens is that, in the antibonding combination with the L′ lone pair, the d_σ_ orbital is effectively pushed up in energy and (through its L′-lone-pair component) interacts in a stabilizing fashion with the metal s-derived LUMO on ML.

The aforementioned π backbonding that favors bending (see Figure 3) shows up in an increased stabilization in the asymmetric Δ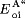 component as the L–M–L angle decreases. To more clearly reveal the role of the orbital interactions within A′′ symmetry, we separate the interaction energy Δ*E*_int_ into the corresponding term 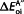 plus the remaining interaction energy Δ*E′*_int_, which combines the other interaction terms comprising electrostatic attraction Δ*V*_elstat_, Pauli repulsion Δ*E*_Pauli_, and the symmetric orbital interactions Δ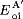:


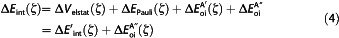
(4)

Thus, the interaction energy is split into two contributions which are both stabilizing along a large part of the energy profiles studied and which vary over a significantly smaller range. Therefore, this decomposition allows us to directly compare the importance of Δ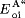 with respect to the combined influence of all other terms, contained in Δ*E′*_int_. The latter contains the aforementioned counteracting and largely canceling terms of strong Pauli repulsion between A′ orbitals and the resulting stabilizing relaxation effect Δ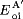.

The results of this alternative decomposition appear in Figure 5, again for the series of palladium complexes Pd(NH_3_)_2_, Pd(PH_3_)_2_ and Pd(CO)_2_. In each of these complexes, bending begins at a certain point to weaken the Δ*E′*_int_ energy term and, at smaller L–M–L angles, makes it eventually repulsive as the Pauli repulsion term becomes dominant (see also Figure 4). Numerical experiments, in which we consider the rigid bending process of a complex in which the metal is removed, show that steric repulsion between ligands does contribute to this repulsion, especially at smaller angles. Thus, direct Pauli repulsion between L and L′ in LM–L′ goes, upon bending from 180° to 90°, from 0.3 to 4.6 kcal mol^−1^ for Pd(NH_3_)_2_ and from 0.4 to 9.0 kcal mol^−1^ for Pd(CO)_2_ (data not shown in Figures). This finding confirms that ligands avoid each other for steric reasons, but it also shows that the effect is small as compared to the overall change in the Δ*E*_int_ curves (see Figure 5). The dominant term that causes Δ*E*_int_ to go up in energy upon bending is the increasing Pauli repulsion that occurs as the L′ lone pair overlaps more effectively with the LM d_σ_ orbital.

In a number of cases, the stabilization upon bending from the asymmetric orbital interactions Δ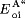 dominates the destabilization from the Δ*E′*_int_ term. These cases are the complexes that adopt nonlinear equilibrium geometries. This 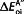 term gains stabilization upon bending LM–L′ because the π*-acceptor orbital on the ligand L′ moves from a position in which it can overlap with a ligand-stabilized LM d_π_ orbital to a more or less pure metal and, thus, up to 1 eV higher-energy d_δ_ orbital (see Table 3), which leads to a more stabilizing donor–acceptor orbital interaction (see Figure 5). The gain in stabilization of Δ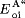 upon bending and, thus, the tendency to bend increases along NH_3_ to PH_3_ to CO. The reason is the increasing π-accepting ability of the ligands as reflected by the energy *ε*(π*) of the ligands′ π* orbital which is lowered from +1.42 to −0.24 to −1.92 eV, respectively (see Table 4). Thus, for Pd(NH_3_)_2_, where π backdonation plays essentially no role, the 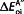 term is stabilized by less than 0.5 kcal mol^−1^ if we go from 180° to 90°. For PH_3_, known as a moderate π-accepting ligand, this energy term is stabilized by 1.5 kcal mol^−1^ from 180° to 90° and, for CO, this stabilization amounts to 2.5 kcal mol^−1^. Thus, in the case of palladium complexes, the energy profile for bending the complexes becomes progressively more flat as the ligands are better π acceptors, but only the carbonyl ligand generates sufficient stabilization through increased π-backbonding in Δ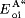 to shift the equilibrium geometry to an angle smaller than 180°.

### Bonding mechanism: Variation of metals

Applying the same analysis along the series Rh(CO)_2_^−^, Pd(CO)_2_ and Ag(CO)_2_^+^, reveals a similar but clearer picture (Figure 6). Along this series of isoelectronic complexes, the equilibrium geometries have L–M–L angles of 130.8°, 155.6° and 180.0°. Similar to the results obtained for the series discussed above, we again find a Δ*E′*_int_ term that is relatively shallow and eventually, at small angles, dominated by the Pauli repulsion. The Δ*E′*_int_ term does not provide additional stabilization upon bending the complex. We do observe, however, a Δ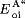 component that, from Rh(CO)_2_^−^ to Pd(CO)_2_ to Ag(CO)_2_^+^, becomes more stabilizing and also gains more stabilization upon bending from 180° to 90°. That is, whereas for Ag(CO)_2_^+^ the 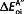 remains constant at a value of −5.4 kcal mol^−1^ as the complex is bent from 180° to 90°; the same component for Pd(CO)_2_ starts already at a more stabilizing value of −15.1 kcal mol^−1^ at 180° and is stabilized more than 2.5 kcal mol^−1^ as the complex is bent to 90°. For Rh(CO)_2_^−^, the effect of the additional stabilization upon bending is strongest, almost 10 kcal mol^−1^, as Δ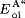 goes from −28.4 kcal mol^−1^ at 180° to −37.3 kcal mol^−1^ at 90°. The mechanism behind this trend is that the donor capability of the metal d orbitals increases as they are pushed up in energy from the cationic AgCO^+^ to the neutral PdCO to the negative RhCO^−^ (see Table 3). This trend of increasing d orbital energies leads to a concomitant strengthening π backdonation and, thus, an increasing energy *difference* in the LM fragment between the pure metal d_δ_ and the ligand-stabilized d_π_ orbitals. Thus, the ”fresh“ d_δ_ orbitals are higher in energy than the ligand-stabilized d_π_ orbitals by 0.21 to 0.96 to 1.65 eV along AgCO^+^, PdCO and RhCO^−^, respectively (see Table 3). Consequently, the LM–L′ complexes benefit progressively along this series from increasing the overlap of L′ π* with the higher-energy d_δ_ orbitals in the bent geometry.

Variation of the metal down a group goes with a less pronounced increase of the L–M–L angle that originates from more subtle changes in the bonding mechanism. The largest variation in bite angle is observed along the group 10 complexes Ni(CO)_2_, Pd(CO)_2_ and Pt(CO)_2_ which show L–M–L angles of 144.5°, 155.6° and 159.0°, respectively (see Table 1). Two factors lie behind this trend: (1) a weakening in π backbonding as the metal orbital energy decreases from nickel 3d to palladium 4d; (2) a steeper increase upon bending in Pauli repulsion between PtCO d_σ_ (that has a large torus due to strong admixture of the relativistically stabilized Pt 6s AO) and the lone pair of the other CO ligand. As shown in Figure 7, the π-backbonding stabilization of Δ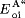 upon bending is indeed stronger for Ni(CO)_2_ than for Pd(CO)_2_ and Pt(CO)_2_. The difference between the latter is small because the greater (more favorable) overlap of the π* orbitals on the ligand with the more extended platinum d orbitals on PtCO compensates for the lower (less favorable) platinum d orbital energy. Figure 7 also shows how the Δ*E′*_int_ term containing the aforementioned Pauli repulsion becomes more rapidly destabilizing at smaller angles for Pt(CO)_2_ than for Ni(CO)_2_ and Pd(CO)_2_. Likewise, in the case of group 9 complexes, the more steeply increasing Pauli repulsion of the ligand lone pair with the large iridium d_σ_ torus pushes the equilibrium L–M–L angle of Ir(CO)_2_^−^ (134.2°) to a larger value than for Rh(CO)_2_^−^ (130.8°; see Table 1). Interestingly, here, the linearization energy Δ*E*_lin_ is nevertheless higher for the less bent Ir(CO)_2_^−^ (13.4 kcal mol^−1^) than for Rh(CO)_2_^−^ (10.2 kcal mol^−1^) because of the more favorable π-backbonding overlap between IrCO^−^ and CO (see Table 1). This illustrates the subtlety of the interplay between the two features in the bonding mechanism.

### Walsh diagrams

**Figure 8:**
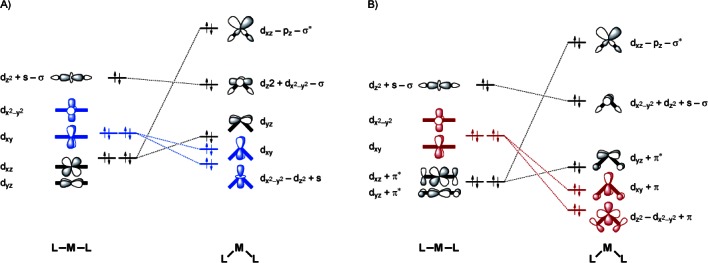
Simplified Walsh diagrams for bending ML_2_ complexes that emerge from our Kohn–Sham MO analyses (+/− indicate bonding/antibonding) A) without and B) with π backbonding.

Based on detailed Kohn–Sham MO analyses of individual complexes, we have constructed generalized Walsh diagrams corresponding to bending the ML_2_ complexes from 180° to 90°. This choice comes down to an alternative perspective on the same problem, and the emerging electronic mechanism, why bending may occur, is fully equivalent to the one obtained in the above analyses based on two interacting fragments LM+L′, namely: Bending ML_2_ to a nonlinear geometry enables ligand π* orbitals (if they are available on L) to overlap with and stabilize metal d orbitals that are not stabilized in the linear arrangement. The spectrum of different bonding situations has been summarized in two simplified diagrams that correspond to two extreme situations: weakly π-accepting ligands (Figure 8 A) and strongly π-accepting ligands (Figure 8 B). In these diagrams, we position the d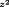 orbital in linear ML_2_ above the other d orbitals, a situation that occurs, for example, for Pd(PH_3_)_2_. The relative position of the d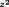 may change, and in some complexes, such as, Rh(NH_3_)_2_^−^, it is located below the other d orbitals. These variations do not affect the essential property of the orbitals, namely, their change in energy upon bending the ML_2_ complex. Furthermore, we speak about weakly π-accepting ligands, not just about (purely) σ-donating ligands, because it turns out that none of our model ligands has negligible π-accepting capability. The resulting Walsh diagrams summarize our results in a more easy to use pictorial manner which, in particular for the situation with strongly π-accepting ligands, is novel.

We first examine the diagram with weakly π-accepting ligands (Figure 8 A). Bending ML_2_ from linear to nonlinear significantly destabilizes the d_xz_ orbital because of turning on overlap with the out-of-phase combination of ligand lone pairs. This effect is related to the overlap between the LM d_σ_ torus and the L′ lone pair in the fragment approach (see above). At small angles, direct ligand–ligand antibonding becomes important. The d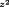 orbital is slightly stabilized in the nonlinear situation due to a decreasing antibonding overlap with the in-phase combination of ligand lone pairs, augmented by admixing with the d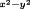 orbital (see a detailed scheme of this intermixing in Figure S1 of the Supporting Information). Note that if our model ligands would have been purely σ donating, the d_xz_, d_yz_ and d_xy_ levels would not be affected by L–M–L bending. Yet, they are, although only slightly so. This is a manifestation of some π backbonding, which is discussed in more detail below for the strongly π-accepting ligands.

In the case of strongly π-accepting orbitals (Figure 8 B), bending ML_2_ from linear to nonlinear still goes with significant destabilization of d_xz_ and slight stabilization of d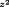 (for the same reasons as discussed above for weakly π-accepting ligands). π Backbonding stabilizes both d_xz_ and d_yz_ in the linear L–M–L arrangement; bending reduces π overlap which causes also d_yz_ to increase in energy. A striking phenomenon in the ML_2_ Walsh diagram with strongly π-accepting ligands is the significant stabilization of the d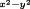 and d_xy_ orbitals that occurs as bending moves ligand π* orbitals in the right orientation for π-accepting overlap with these orbitals. The resulting stabilization, if strong enough, can overcome the destabilization of the d_xz_ orbital and accounts for the observed bent complexes described in this work. This effect is related to the overlap between the LM d_δ_ orbital and the L′ π* in the fragment approach (see above). The same effect also nicely accounts for the nonlinear structures observed in earlier studies for d^0^ metal complexes with π-*donating* ligands.^27^–^31^ For these complexes, a π-bonding mechanism has been proposed in which bending is favorable because it effectively increases the number of d orbitals that have non-zero overlap with the π-donating orbitals on the ligands.^28^

## Conclusion

Dicoordinated d^10^-transition-metal complexes ML_2_ can very well adopt nonlinear geometries with bite angles that deviate significantly from the usually expected 180°. This follows from our relativistic density functional theory (DFT) computations on a broad range of archetypal d^10^-ML_2_ model systems. The smallest bite angle encountered in our exploration among 27 model systems amounts to 128.6° for Co(CO)_2_^−^.

Nonlinear geometries appear to be a direct consequence of π backbonding. The geometry of d^10^-ML_2_ complexes results from two opposing features in the bonding mechanism, which we have analyzed in terms of the interaction between ML and L as a function of the L–M–L angle using quantitative molecular orbital (MO) theory and energy decomposition analyses: Bending destabilizes the interaction Δ*E*_int_ between ML and L through increasing steric (Pauli) repulsion between the ligands’ lone-pair orbital lobes as well as a destabilization, by the latter, of the ML d_σ_ hybrid orbital; however, bending *can also stabilize* Δ*E*_int_ because of enhanced π backdonation. The reason is that the π-accepor orbital on the ligand L (e.g., CO π*) interacts in the linear arrangement with an already stabilized ML d_π_ hybrid orbital, whereas in the bent geometry, it enters into a more favorable donor–acceptor orbital interaction with an unstabilized, that is, higher-energy metal d_δ_ orbital.

Our analyses complement the existing text-book Walsh diagram for bending ML_2_ complexes^8^ with a variant that includes metal–ligand π backbonding. Our findings also contribute to a more rational design of catalytically active and selective ML_2_ complexes.^1^, ^32^

## Supplementary material

As a service to our authors and readers, this journal provides supporting information supplied by the authors. Such materials are peer reviewed and may be re-organized for online delivery, but are not copy-edited or typeset. Technical support issues arising from supporting information (other than missing files) should be addressed to the authors.
