# Supplementary material for: Nonlinear d10-ML2 Transition-Metal Complexes
Source: ChemistryOpen. 2013 May 6;2(3):106–14. doi: 10.1002/open.201300009 (PMC3703815; doi:10.1002/open.201300009)
Supplement: Supplementary file 1 [file open0002-0106-SD1.pdf]

ChemistryOPEN

Supporting Information

© 2013 The Authors. Published by Wiley-VCH Verlag GmbH & Co. KGaA, Weinheim

Nonlinear d^{10}-ML_2 Transition-Metal Complexes

Lando P. Wolters[a] and F. Matthias Bickelhaupt*[a, b]

open_201300009_sm_miscellaneous_information.pdf

Content

Figure S1. Molecular orbital diagram for the intermixing of $d_{x^2-y^2}$ and d_{z^2} with ligand orbitals.

Table S1. Cartesian coordinates and ADF total bonding energies of all species in this studies, computed at ZORA-BLYP/TZ2P.

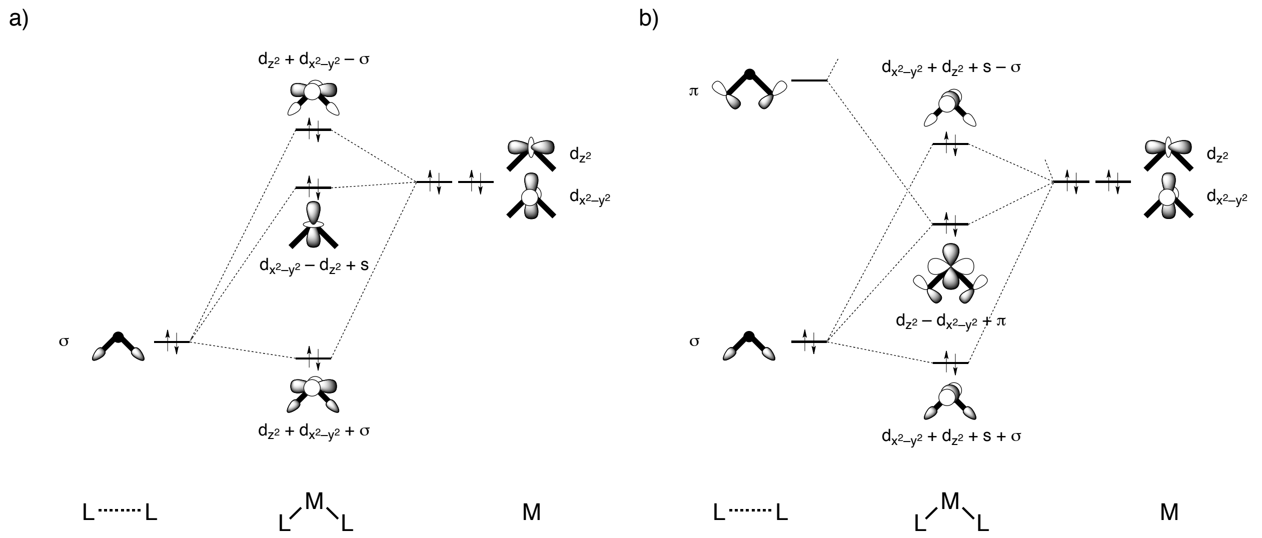

Figure S1. Molecular orbital diagram for the mixing of ligand orbitals with the metal $d_{x^2-y^2}$ and d_{z^2} orbitals, without (a) and with π-backbonding (b). The lowest-energy molecular orbital that results from these interactions is not shown in the Walsh diagrams in Figure 8 of the main text.

Table S1. Cartesian coordinates (in Å) and ADF total bonding energies (in kcal mol^{-1}) of all species in this studies, computed at ZORA-BLYP/TZ2P.

Co^- (s^0d^{10})			[+165.77]	Ni ($d^{10}s^0$)			[+39.31]	Cu^+ ($d^{10}s^0$)			[+193.87]
Rh^- (s^0d^{10})			[−2.54]	Pd ($d^{10}s^0$)			[0.00]	Ag^+ ($d^{10}s^0$)			[+181.66]
Ir^- (s^0d^{10})			[+16.50]	Pt ($d^{10}s^0$)			[+1.78]	Au^+ ($d^{10}s^0$)			[+217.95]
NH_3			[-434.00]	PH_3			[-344.15]	CO			[-332.64]
N	0.00000000	0.00000000	0.00000000	P	0.00000000	0.00000000	0.00000000	C	0.00000000	0.00000000	0.00000000
H	-0.94669654	0.00000000	0.38465769	H	-1.19602474	0.00000000	0.78247765	O	0.00000000	0.00000000	1.13685222
H	0.47334827	-0.81986325	0.38465769	H	0.59801237	-1.03578781	0.78247765				
H	0.47334827	0.81986325	0.38465769	H	0.59801237	1.03578781	0.78247765				
$CoNH_3^-$			[−485.36]	$NiNH_3$			[−471.65]	$CuNH_3^+$			[−310.16]
Co	0.00000000	0.00000000	0.00000000	Ni	0.00000000	0.00000000	0.00000000	Cu	0.00000000	0.00000000	0.00000000
N	0.00000000	0.00000000	1.84472160	N	0.00000000	0.00000000	1.82738202	N	0.00000000	0.00000000	1.91128850
H	-0.98308157	0.00000000	2.21082317	H	-0.96714131	0.00000000	2.18222096	H	-0.95585541	0.00000000	2.28610213
H	0.49154078	-0.85137361	2.21082317	H	0.48357066	-0.83756895	2.18222096	H	0.47792770	-0.82779507	2.28610213
H	0.49154078	0.85137361	2.21082317	H	0.48357066	0.83756895	2.18222096	H	0.47792770	0.82779507	2.28610213
$CoPH_3^-$			[−418.94]	$NiPH_3$			[−392.88]	$CuPH_3^+$			[−218.93]
Co	0.00000000	0.00000000	0.00000000	Ni	0.00000000	0.00000000	0.00000000	Cu	0.00000000	0.00000000	0.00000000
P	0.00000000	0.00000000	1.97140876	P	0.00000000	0.00000000	1.97913351	P	0.00000000	0.00000000	2.16270628
H	-1.24067845	0.00000000	2.75755377	H	-1.23510116	0.00000000	2.70154490	H	-1.28006834	0.00000000	2.75724511
H	0.62033923	-1.07445906	2.75755377	H	0.61755058	-1.06962898	2.70154490	H	0.64003417	-1.10857170	2.75724511
H	0.62033923	1.07445906	2.75755377	H	0.61755058	1.06962898	2.70154490	H	0.64003417	1.10857170	2.75724511
$CoCO^-$			[−447.44]	NiCO			[−402.63]	$CuCO^+$			[−188.94]
Co	0.00000000	0.00000000	0.00000000	Ni	0.00000000	0.00000000	0.00000000	Cu	0.00000000	0.00000000	0.00000000
C	0.00000000	0.00000000	1.62957249	C	0.00000000	0.00000000	1.66308816	C	0.00000000	0.00000000	1.83299104
O	0.00000000	0.00000000	2.83960023	O	0.00000000	0.00000000	2.83029178	O	0.00000000	0.00000000	2.96162145
$Co(NH_3)_2^-$			[−943.31]	$Ni(NH_3)_2$			[−941.85]	$Cu(NH_3)_2^+$			[−805.22]
Co	0.00000000	0.00000000	0.00000000	Ni	0.00000000	0.00000000	0.00000000	Cu	0.00000000	0.00000000	0.00000000
N	0.00000000	0.00000000	1.90840596	N	0.00000000	0.00000000	1.88827279	N	0.00000000	0.00000000	1.91905807
H	-0.97480885	0.00000000	2.27682692	H	-0.96079533	0.00000000	2.25607197	H	-0.95041886	0.00000000	2.30550740
H	0.48740442	-0.84420923	2.27682692	H	0.48039766	-0.83207316	2.25607197	H	0.47520943	-0.82308687	2.30550740
H	0.48740442	0.84420923	2.27682692	H	0.48039766	0.83207316	2.25607197	H	0.47520943	0.82308687	2.30550740
N	0.00000000	0.00000000	-1.90840596	N	0.00000000	0.00000000	-1.88827279	N	0.00000000	0.00000000	-1.91905807
H	-0.97480885	0.00000000	-2.27682692	H	-0.96079533	0.00000000	-2.25607197	H	-0.95041886	0.00000000	-2.30550740
H	0.48740442	-0.84420923	-2.27682692	H	0.48039766	-0.83207316	-2.25607197	H	0.47520943	-0.82308687	-2.30550740
H	0.48740442	0.84420923	-2.27682692	H	0.48039766	0.83207316	-2.25607197	H	0.47520943	0.82308687	-2.30550740
$Co(PH_3)_2^-$			[−811.26]	$Ni(PH_3)_2$			[−773.33]	$Cu(PH_3)_2^+$			[−611.10]
Co	0.00000000	0.00000000	0.00000000	Ni	0.00000000	0.00000000	0.00000000	Cu	0.00000000	0.00000000	0.00000000
P	-0.97752782	1.01184533	1.49365044	P	0.00000000	0.00000000	-2.10784490	P	-0.38528798	0.67698120	2.09150605
H	-1.11907450	2.45835960	1.41490071	H	1.05863644	0.61120403	-2.84643928	H	-1.08461974	1.89432190	2.25350506
H	-2.39479479	0.77177971	1.72238394	H	0.00000000	-1.22240807	-2.84643928	H	-1.14737797	-0.18226472	2.91499404
H	-0.77231381	1.12341108	2.95684115	H	-1.05863644	0.61120403	-2.84643928	H	0.74307993	0.90412897	2.91148048
P	-0.31951389	-0.12789065	-2.02289604	P	0.00000000	0.00000000	2.10784490	P	0.38409687	-0.67490308	-2.09239202
H	-0.32426359	1.08175315	-2.83244813	H	-1.05863644	-0.61120403	2.84643928	H	-0.09308578	0.15481418	-3.13218162
H	0.36517452	-0.84708240	-3.12263904	H	1.05863644	-0.61120403	2.84643928	H	1.73405913	-0.83841532	-2.47726661
H	-1.60023133	-0.60448938	-2.52411944	H	0.00000000	1.22240807	2.84643928	H	-0.15743722	-1.92304043	-2.47459288
$Co(CO)_2^-$			[−856.41]	$Ni(CO)_2$			[−783.89]	$Cu(CO)_2^+$			[−566.58]
Co	0.00000000	0.00000000	0.00000000	Ni	0.00000000	0.00000000	-0.63774400	Cu	0.00000000	0.00000000	0.00000000
C	-1.54472129	0.00000000	0.74408161	C	0.00000000	1.68092377	-0.09904975	C	0.00000000	0.00000000	1.88134839
O	-2.45745522	0.00000000	1.51664055	O	0.00000000	2.71507961	0.41787784	O	0.00000000	0.00000000	3.00883641
C	1.54472129	0.00000000	0.74408161	C	0.00000000	-1.68092377	-0.09904975	C	0.00000000	0.00000000	-1.88134839
O	2.45745522	0.00000000	1.51664055	O	0.00000000	-2.71507961	0.41787784	O	0.00000000	0.00000000	-3.00883641
$RhNH_3^-$			[−492.01]	$PdNH_3$			[−455.54]	$AgNH_3^+$			[−301.00]
Rh	0.00000000	0.00000000	0.00000000	Pd	0.00000000	0.00000000	0.00000000	Ag	0.00000000	0.00000000	0.00000000
N	0.00000000	0.00000000	2.00144073	N	0.00000000	0.00000000	2.11511242	N	0.00000000	0.00000000	2.21161759
H	-0.97756141	0.00000000	2.34442524	H	-0.95824704	0.00000000	2.47004875	H	-0.95405286	0.00000000	2.58318457
H	0.48878070	-0.84659301	2.34442524	H	0.47912352	-0.82986628	2.47004875	H	0.47702643	-0.82623401	2.58318457
H	0.48878070	0.84659301	2.34442524	H	0.47912352	0.82986628	2.47004875	H	0.47702643	0.82623401	2.58318457
$RhPH_3^-$			[−436.61]	$PdPH_3$			[−383.54]	$AgPH_3^+$			[−210.38]
Rh	0.00000000	0.00000000	0.00000000	Pd	0.00000000	0.00000000	0.00000000	Ag	0.00000000	0.00000000	0.00000000
P	0.00000000	0.00000000	2.06845580	P	0.00000000	0.00000000	2.17205185	P	0.00000000	0.00000000	2.41536382
H	-1.21490445	0.00000000	2.86794039	H	-1.22193216	0.00000000	2.90349549	H	-1.27053687	0.00000000	3.02947812
H	0.60745222	-1.05213812	2.86794039	H	0.61096608	-1.05822429	2.90349549	H	0.63526843	-1.10031721	3.02947812
H	0.60745222	1.05213812	2.86794039	H	0.61096608	1.05822429	2.90349549	H	0.63526843	1.10031721	3.02947812
$RhCO^-$			[−457.15]	PdCO			[−380.02]	$AgCO^+$			[−179.42]
Rh	0.00000000	0.00000000	0.00000000	Pd	0.00000000	0.00000000	0.00000000	Ag	0.00000000	0.00000000	0.00000000
C	0.00000000	0.00000000	1.74959588	C	0.00000000	0.00000000	1.86089238	C	0.00000000	0.00000000	2.13655348
O	0.00000000	0.00000000	2.94609194	O	0.00000000	0.00000000	3.01665453	O	0.00000000	0.00000000	3.26266178
$Rh(NH_3)_2^-$			[−948.6442]	$Pd(NH_3)_2$			[−918.12]	$Ag(NH_3)_2^+$			[−780.21]
Rh	0.00000000	0.00000000	0.00000000	Pd	0.00000000	0.00000000	0.00000000	Ag	0.00000000	0.00000000	0.00000000
N	0.00000000	0.00000000	2.08880988	N	0.00000000	0.00000000	2.10562276	N	0.00000000	0.00000000	2.17159509
H	-0.96758966	0.00000000	2.44879126	H	-0.95714408	0.00000000	2.46814211	H	-0.95046138	0.00000000	2.55153578
H	0.48379483	-0.83795723	2.44879126	H	0.47857204	-0.82891109	2.46814211	H	0.47523069	-0.82312370	2.55153578
H	0.48379483	0.83795723	2.44879126	H	0.47857204	0.82891109	2.46814211	H	0.47523069	0.82312370	2.55153578
N	0.00000000	0.00000000	-2.08880988	N	0.00000000	0.00000000	-2.10562276	N	0.00000000	0.00000000	-2.17159509
H	-0.96758966	0.00000000	-2.44879126	H	-0.95714408	0.00000000	-2.46814211	H	-0.95046138	0.00000000	-2.55153578
H	0.48379483	-0.83795723	-2.44879126	H	0.47857204	-0.82891109	-2.46814211	H	0.47523069	-0.82312370	-2.55153578
H	0.48379483	0.83795723	-2.44879126	H	0.47857204	0.82891109	-2.46814211	H	0.47523069	0.82312370	-2.55153578
$Rh(PH_3)_2^-$			[−818.97]	$Pd(PH_3)_2$			[−756.30]	$Ag(PH_3)_2^+$			[−592.59]
Rh	0.00000000	0.00000000	0.00000000	Pd	0.00000000	0.00000000	0.00000000	Ag	0.00000000	0.00000000	0.00000000
P	0.00000000	0.00000000	-2.21815534	P	0.00000000	0.00000000	2.28723821	P	0.00000000	0.00000000	-2.44257985
H	0.60072639	-1.04048863	-3.02309974	H	0.60880654	-1.05448386	3.02445701	H	0.62724530	1.08642073	-3.09221865
H	-1.20145278	0.00000000	-3.02309974	H	0.60880654	1.05448386	3.02445701	H	0.62724530	-1.08642073	-3.09221865
H	0.60072639	1.04048863	-3.02309974	H	-1.21761309	0.00000000	3.02445701	H	-1.25449060	0.00000000	-3.09221865
P	0.00000000	0.00000000	2.21815534	P	0.00000000	0.00000000	-2.28723821	P	0.00000000	0.00000000	2.44257985
H	0.60072639	-1.04048863	3.02309974	H	0.60880654	1.05448386	-3.02445701	H	0.62724530	1.08642073	3.09221865
H	0.60072639	1.04048863	3.02309974	H	-1.21761309	0.00000000	-3.02445701	H	-1.25449060	0.00000000	3.09221865
H	-1.20145278	0.00000000	3.02309974	H	0.60880654	1.05448386	-3.02445701	H	0.62724530	-1.08642073	3.09221865

$Rh(CO)_2^-$			[-847.94]
Rh	0.00000000	0.00000000	0.00000000
C	-1.69616138	0.00000000	0.77677342
O	-2.59107157	0.00000000	1.55525490
C	1.69616138	0.00000000	0.77677342
O	2.59107157	0.00000000	1.55525490

$IrNH_3^-$			[-502.46]
Ir	0.00000000	0.00000000	0.00000000
N	0.00000000	0.00000000	1.96729882
H	-0.97665900	0.00000000	2.33397792
H	0.48832950	-0.84581150	2.33397792
H	0.48832950	0.84581150	2.33397792

$IrPH_3^-$			[-454.17]
Ir	0.00000000	0.00000000	0.00000000
P	0.00000000	0.00000000	2.05647914
H	0.62000459	-1.07387946	2.81727128
H	-1.24000919	0.00000000	2.81727128
H	0.62000459	1.07387946	2.81727128

$IrCO^-$			[-482.43]
Ir	0.00000000	0.00000000	0.00000000
C	0.00000000	0.00000000	1.73360818
O	0.00000000	0.00000000	2.93745671

$Ir(NH_3)_2^-$			[-960.03]
Ir	0.00000000	0.00000000	0.00000000
N	0.00000000	0.00000000	2.07058451
H	-0.96629572	0.00000000	2.44321743
H	0.48314786	-0.83683664	2.44321743
H	0.48314786	0.83683664	2.44321743
N	0.00000000	0.00000000	-2.07058451
H	-0.96629572	0.00000000	-2.44321743
H	0.48314786	-0.83683664	-2.44321743
H	0.48314786	0.83683664	-2.44321743

$Ir(PH_3)_2^-$			[-842.44]
Ir	0.00000000	0.00000000	0.69592050
P	0.00000000	2.08321807	1.36978823
H	0.00000000	3.37212829	0.65703177
H	-1.06646209	2.52986440	2.23124490
H	1.06646209	2.52986440	2.23124490
P	0.00000000	-2.08321807	1.36978823
H	0.00000000	-3.37212829	0.65703177
H	1.06646209	-2.52986440	2.23124490
H	-1.06646209	-2.52986440	2.23124490

$Ir(CO)_2^-$			[-881.34]
Ir	0.00000000	0.00000000	0.00000000
C	-1.70750444	0.00000000	0.72174648
O	-2.63203751	0.00000000	1.47280574
C	1.70750444	0.00000000	0.72174648
O	2.63203751	0.00000000	1.47280574

$Co(PH_3)_2^-$ [D_{3h}]			[−804.95]
Co	0.00000000	0.00000000	0.00000000
P	0.00000000	0.00000000	2.08536773
H	1.05421861	-0.60865340	2.88604139
H	0.00000000	1.21730680	2.88604139
H	-1.05421861	-0.60865340	2.88604139
P	0.00000000	0.00000000	-2.08536773
H	-1.05421861	0.60865340	-2.88604139
H	1.05421861	0.60865340	-2.88604139
H	0.00000000	-1.21730680	-2.88604139

$Co(CO)_2^-$ [$D_{\infty h}$]			[-836.52]
Co	0.00000000	0.00000000	0.00000000
C	0.00000000	0.00000000	1.76467991
O	0.00000000	0.00000000	2.95000321
C	0.00000000	0.00000000	-1.76467991
O	0.00000000	0.00000000	-2.95000321

$Rh(PH_3)_2^-$ [D_{3h}]			[-816.96]
Rh	0.00000000	0.00000000	0.00000000
P	0.00000000	0.00000000	-2.21815534
H	0.60072639	-1.04048863	-3.02309974
H	-1.20145278	0.00000000	-3.02309974
H	0.60072639	1.04048863	-3.02309974
P	0.00000000	0.00000000	2.21815534
H	0.60072639	-1.04048863	3.02309974
H	0.60072639	1.04048863	3.02309974
H	-1.20145278	0.00000000	3.0230997

$Rh(CO)_2^-$ [$D_{\infty h}$]			[-837.77]
Rh	0.00000000	0.00000000	0.00000000
C	0.00000000	0.00000000	1.90928797
O	0.00000000	0.00000000	3.08218877
C	0.00000000	0.00000000	-1.90928797
O	0.00000000	0.00000000	-3.08218877

$Ir(PH_3)_2^-$ [D_{3h}]			[−-840.07]
Ir	0.00000000	0.00000000	0.00000000
P	0.00000000	0.00000000	2.21076208
H	-0.60828651	1.05358313	2.99131941
H	-0.60828651	-1.05358313	2.99131941
H	1.21657301	0.00000000	2.99131941
P	0.00000000	0.00000000	-2.21076208
H	-0.60828651	1.05358313	-2.99131941
H	1.21657301	0.00000000	-2.99131941
H	-0.60828651	-1.05358313	-2.99131941

$Ir(CO)_2^-$ [$D_{\infty h}$]			[-867.95]
Ir	0.00000000	0.00000000	0.00000000
C	0.00000000	0.00000000	1.90083961
O	0.00000000	0.00000000	3.07682715
C	0.00000000	0.00000000	-1.90083961
O	0.00000000	0.00000000	-3.07682715

$Pd(CO)_2$			[-747.40]
Pd	0.00000000	0.00000000	0.00000000
C	0.00000000	-1.90528693	-0.41228329
O	0.00000000	-2.99823353	-0.76339230
C	0.00000000	1.90528693	-0.41228329
O	0.00000000	2.99823353	-0.76339230

$PtNH_3$			[-482.26]
Pt	0.00000000	0.00000000	0.00000000
N	0.00000000	0.00000000	1.98078444
H	-0.96779494	0.00000000	2.32263285
H	0.48389747	-0.83813500	2.32263285
H	0.48389747	0.83813500	2.32263285

$PtPH_3$			[-419.61]
Pt	0.00000000	0.00000000	0.00000000
P	0.00000000	0.00000000	2.09525493
H	-1.24947482	0.00000000	2.77273058
H	0.62473741	-1.08207694	2.77273058
H	0.62473741	1.08207694	2.77273058

$PtCO$			[-418.78]
Pt	0.00000000	0.00000000	0.00000000
C	0.00000000	0.00000000	1.77640737
O	0.00000000	0.00000000	2.93799331

$Pt(NH_3)_2$			[-957.89]
Pt	0.00000000	0.00000000	0.00000000
N	0.00000000	0.00000000	2.06082030
H	0.47946523	-0.83045814	2.42208863
H	0.47946523	0.83045814	2.42208863
H	-0.95893046	0.00000000	2.42208863
N	0.00000000	0.00000000	-2.06082030
H	0.47946523	-0.83045814	-2.42208863
H	-0.95893046	0.00000000	-2.42208863
H	0.47946523	0.83045814	-2.42208863

$Pt(PH_3)_2$			[-802.43]
Pt	0.00000000	0.00000000	0.00000000
P	0.00000000	0.00000000	2.24913921
H	0.61537140	-1.06585453	2.96140633
H	0.61537140	1.06585453	2.96140633
H	-1.23074280	0.00000000	2.96140633
P	0.00000000	0.00000000	-2.24913921
H	0.61537140	-1.06585453	-2.96140633
H	-1.23074280	0.00000000	-2.96140633
H	0.61537140	1.06585453	-2.96140633

$Pt(CO)_2$			[-798.54]
Pt	0.00000000	0.00000000	-0.87440400
C	0.00000000	-1.87943075	-0.52609898
O	0.00000000	-2.97450638	-0.17383272
C	0.00000000	1.87943075	-0.52609898
O	0.00000000	2.97450638	-0.17383272

$Ni(CO)_2$ [$D_{\infty h}$]			[-781.02]
Ni	0.00000000	0.00000000	0.00000000
C	0.00000000	0.00000000	1.78186588
O	0.00000000	0.00000000	2.93437773
C	0.00000000	0.00000000	-1.78186588
O	0.00000000	0.00000000	-2.93437773

$Pd(CO)_2$ [$D_{\infty h}$]			[-746.91]
Pd	0.00000000	0.00000000	0.00000000
C	0.00000000	0.00000000	1.95675205
O	0.00000000	0.00000000	3.10249810
C	0.00000000	0.00000000	-1.95675205
O	0.00000000	0.00000000	-3.10249810

$Pt(CO)_2$ [$D_{\infty h}$]			[-797.94]
Pt	0.00000000	0.00000000	0.00000000
C	0.00000000	0.00000000	1.91991082
O	0.00000000	0.00000000	3.06774725
C	0.00000000	0.00000000	-1.91991082
O	0.00000000	0.00000000	-3.06774725

$Ag(CO)_2^+$			[-542.73]
Ag	0.00000000	0.00000000	0.00000000
C	0.00000000	0.00000000	2.11270784
O	0.00000000	0.00000000	3.23894702
C	0.00000000	0.00000000	-2.11270784
O	0.00000000	0.00000000	-3.23894702

$AuNH_3^+$			[-287.41]
Au	0.00000000	0.00000000	0.00000000
N	0.00000000	0.00000000	2.08495377
H	-0.96477816	0.00000000	2.43335078
H	0.48238908	-0.83552240	2.43335078
H	0.48238908	0.83552240	2.43335078

$AuPH_3^+$			[-210.40]
Au	0.00000000	0.00000000	0.00000000
P	0.00000000	0.00000000	2.23958510
H	-1.29520069	0.00000000	2.79520516
H	0.64760035	-1.12167670	2.79520516
H	0.64760035	1.12167670	2.79520516

$AuCO^+$			[-169.73]
Au	0.00000000	0.00000000	0.00000000
C	0.00000000	0.00000000	1.92662084
O	0.00000000	0.00000000	3.05607061

$Au(NH_3)_2^+$			[-786.05]
Au	0.00000000	0.00000000	0.00000000
N	0.00000000	0.00000000	2.08804100
H	-0.95533348	0.00000000	2.45867078
H	0.47766674	-0.82734306	2.45867078
H	0.47766674	0.82734306	2.45867078
N	0.00000000	0.00000000	-2.08804100
H	-0.95533348	0.00000000	-2.45867078
H	0.47766674	-0.82734306	-2.45867078
H	0.47766674	0.82734306	-2.45867078

$Au(PH_3)_2^+$			[-607.15]
Au	0.00000000	0.00000000	0.00000000
P	0.00000000	0.00000000	2.35097967
H	-0.63323787	1.09680017	2.97378831
H	-0.63323787	-1.09680017	2.97378831
H	1.26647574	0.00000000	2.97378831
P	0.00000000	0.00000000	-2.35097967
H	-0.63323787	1.09680017	-2.97378831
H	1.26647574	0.00000000	-2.97378831
H	-0.63323787	-1.09680017	-2.97378831

$Au(CO)_2^+$			[-552.55]
Au	0.00000000	0.00000000	0.00000000
C	0.00000000	0.00000000	2.00241463
O	0.00000000	0.00000000	3.12982845
C	0.00000000	0.00000000	-2.00241463
O	0.00000000	0.00000000	-3.12982845
